# Prognostic Nomograms for Predicting Overall Survival and Cancer-Specific Survival of Patients with Major Salivary Gland Mucoepidermoid Carcinoma

**DOI:** 10.7150/jca.27992

**Published:** 2019-07-22

**Authors:** Jia-Qian Hu, Peng-Cheng Yu, Xiao Shi, Wan-Lin Liu, Ting-Ting Zhang, Bo-Wen Lei, Nai-Si Huang, Wei-Bo Xu, Li-Tao Han, Ben Ma, Tian Liao, Wen-Jun Wei, Yu Wang, Zhong-Wu Lu, Yu-Long Wang, Qing-Hai Ji

**Affiliations:** 1 Department of Head and Neck Surgery, Fudan University Shanghai Cancer Center, Shanghai, 200032, China; 2 Department of Oncology, Shanghai Medical College, Fudan University, Shanghai, 200032, China

**Keywords:** Major salivary gland, Mucoepidermoid carcinoma, Nomogram, Overall survival, Cancer-specific survival, C-index.

## Abstract

**Background:** The aim of this study was to develop and validate prognostic nomograms predicting overall (OS) and cancer-specific survival (CSS) of patients with major salivary gland (MaSG) mucoepidermoid carcinoma (MEC).

**Methods:** 1398 MaSG-MEC patients were identified from the Surveillance, Epidemiology and End Results (SEER) database. They were randomly and equally divided into a training cohort (n=699) and a validation cohort (n=699). The best subsets of covariates were identified to develop nomograms predicting OS and CSS based on the smallest Akaike Information Criterion (AIC) value in the multivariate Cox models. The nomograms were internally and externally validated by the bootstrap resampling method. The predictive ability was evaluated by Harrell's Concordance Index (C-index).

**Results:** For the training cohort, eight (age at diagnosis, tumor grade, primary site, surgery, radiation, T, N and M classification) and seven predictors (all the above factors except primary site) were selected to create the nomograms estimating the 3- and 5- year OS and CSS, respectively. C-index indicated better predictive performance of the nomograms than the 7th AJCC staging system, which was confirmed by both internal (*via* the training cohort: OS: 0.888 *vs* 0.785, CSS: 0.938 *vs* 0.821) and external validation (*via* the validation cohort: OS: 0.844 *vs* 0.743, CSS: 0.882 *vs* 0.787). The calibration plots also revealed good agreements between the nomogram-based prediction and observed survival.

**Conclusions:** We have proposed and validated the nomograms predicting OS and CSS of MaSG-MEC. They are proved to be of higher predictive value than the AJCC staging system and may be adopted in future clinical practice.

## Introduction

Major salivary gland carcinoma (MaSGC) is a group of relatively rare diseases accounting for less than 5% of all head and neck cancers.^1^, ^2^ Regardless of its low incidence, MaSGC is composed of as many as 23 histologic subtypes according to the WHO classification,^3^ which is very different from the majority of head and neck cancers that predominated by squamous cell carcinoma. Among these heterogeneous histotypes, mucoepidermoid carcinoma (MEC) is the most common one, representing 30%-40% of all major salivary gland malignancies.^4^ MEC tumor is named after its three composing cell types in widely varying proportions: intermediate cells, mucous cells, and epidermoid cells. The combination of these three cell types histologically generates low, intermediate and high grade MEC. As a matter of fact, extensive studies have identified tumor grade as an important prognostic factor for MEC 5-7. However, the definition of low and high grade MEC remains controversial, challenging the stratification and treatment for those patients.

Currently, the American Joint Committee on Cancer (AJCC) staging system has been widely used to predict the survival of MaSG-MEC based on the tumor size or the extent of invasion (T), nodal involvement (N) and distant metastasis (M). A major flaw in this system is that it fails to include tumor grade as a parameter in MEC prognosis, which undisputedly compromises its utility. In addition, other clinicopathologic factors such as patients' age, primary site, and treatment, are also likely to play a role in the prognosis of MaSG-MEC. Taking account of the combined effects of these various prognostic factors, the heterogeneity of outcomes cannot be accurately estimated by the AJCC system. Consequently, a comprehensive predictive model incorporating more prognostic information needs to be proposed.

Integrating several important factors into an intuitive graph with a user-friendly interface, nomograms have been proposed as an alternative to the AJCC classification to quantify risks and estimate prognosis of many cancer types.^8^-^11^ Therefore, in this study, we aimed to develop effective nomograms based on a large population-based dataset to estimate overall survival (OS) and cancer-specific survival (CSS) of MaSG-MEC patients for the first time, in order to provide practical information to help clinicians make individualized recommendations in clinical practice.

## Materials and Methods

### Data source

Patients' data were extracted from the Surveillance, Epidemiology, and End Results (SEER) database which collects information of cancer patients in 18 registries across the nation and covers 28% of total population in the United States. The SEER program collects patients' data on demographics, site of the primary lesion, morphology, tumor grade, TNM classification, treatment and vital status. The mortality information is updated each year by Data Analysis and Interpretation Branch of the National Cancer Institute (NCI). (http://www.seer.cancer.gov)

### Inclusion criteria

SEER*Stat 8.3.4 was applied to extract data from the database with the following inclusion criteria: **1)** Diagnosed with primary mucoepidermoid carcinoma; **2)** Primary sites were limited according to International Classification of Diseases for Oncology, 3rd edition (ICD-O-3) codes: C07.9-Parotid gland, C08.0-Submandibular gland and C08.1-Sublingual gland; **3)** Diagnosed between January 1, 2004 and December 31, 2013; **4)** Known T, N and M staging, **5)** Known tumor grade; **6)** Known information on surgery and radiation.

Surgical treatment of MaSG-MEC includes resection of the primary tumor and neck dissection. Nevertheless, detailed information as to the surgical extent of neck dissection cannot be acquired in SEER database, preventing us from further analysis. Besides, although the number of patients who received partial or total parotidectomy is available in SEER, this parameter is not essential in the context of MaSG-MEC. To be more specific, partial resection is not an option in the treatment of tumors originated from submandibular or sublingual glands. Therefore, in this study, surgery means the patient had received surgical treatment of the primary site, regardless of specific ways.

Of note, the seventh edition of the AJCC staging system was adopted in this study because extranodal extension, an important factor of the eighth AJCC N classification, was not available to most cases in SEER database.

### Study design and statistical analysis

The clinicopathological factors extracted from the SEER database included age at diagnosis, sex, primary site, tumor grade, surgery, radiation, T, N and M classification, survival time and vital status.

The overall patients were randomly and equally divided into a training cohort and a validation cohort. The training cohort was used to conduct survival analysis and develop nomograms while the validation cohort was used to perform external validation of the established nomograms.

Overall survival (OS), as one of our major endpoints of interest, was measured as the interval from diagnosis to death or the cutoff date of follow-up. Cancer-specific survival (CSS) was another endpoint of interest. We measured cancer-specific survival as the interval from diagnosis to the date of death resulting from MaSGC-MEC or the date of the last follow-up.

### Development of the nomograms

Using the patients in the training cohort, the covariates included in the multivariate Cox proportional hazards models were identified by a backward stepwise method based on the smallest Akaike information criterion (AIC) value, which indicated the minimal loss of prognostic information.^12^, ^13^ The nomograms integrating the best subsets of clinicopathological variables in the multivariate analyses were developed to predict the 3- and 5-year OS and CSS.

### Validation of the nomograms

By means of the bootstrap method with 1000 resamples, the predictive performance of the nomograms was assessed in both the training cohort (internal validation) and the validation cohort (external validation). Harrell's concordance index (C-index) was calculated to evaluate the predictive ability of the nomograms. Ranging from 0.5 to 1.0,^14^ C-index reflects the concordance between the prediction and observed outcomes. Generally speaking, a C-index value over 0.7 indicates a good match.^8^ Calibration plots were also performed to evaluate the predictive accuracy. The training and validation cohorts were randomly divided into five prognostic groups with equal sample size for internal and external validation, respectively. Each calibration plot consists of a diagonal line and an irregular curve.^15^ The irregular curve includes five points representing the average nomogram-predicted survival of the five groups, while the diagonal line indicates a perfect match between nomogram-predicted survival (x-axis) and observed survival (y-axis). The closer the irregular curve is to the diagonal line, the more accurately the nomogram predicts.

IBM SPSS, version 19 (SPSS Inc, Chicago, IL, USA) was used to conduct the multivariate Cox proportional hazards analyses. The *rms* 16 package in R program (version 3.3.1) were used to perform the nomogram establishment and validation (http://www.r-project.org/). A two-sided *P* value less than 0.05 was considered to be statistically significant.

## Results

### Baseline characteristics

In total, 1398 patients diagnosed with primary MaSG-MEC were identified from the Surveillance, Epidemiology, and End Results (SEER) database and were randomly and equally divided into a training cohort (n=699) and a validation cohort (n=699). All patients in our research were actively followed up before the date of death or the cutoff follow-up date. The baseline characteristics of these patients are listed in **Table 1.**

For the training cohort, the median follow-up time was 56 months (range: 1-131 months). Of these 699 patients, 365 (52.2%) were male and 622 (89.0%) had primary tumors from parotid gland. 618 patients (88.4%) underwent surgery and 336 patients (48.1%) received radiotherapy, respectively. By the end of the last follow-up, 80 patients (11.4%) had died from MEC and 59 patients (8.4%) had died from other causes.

For the 699 patients in the validation cohort, the median follow-up time was 50 months (range: 1 to 131 months). Demographics and tumor characteristics of these patients are also summarized in **Table 1.**

### Establishment of the prognostic nomograms for OS and CSS

The smallest Akaike information criterion (AIC) value occurred when we incorporated eight variables (age at diagnosis, tumor grade, primary site, surgery, radiation, T, N and M classification) into the multivariate Cox regression model for OS (AIC=1405.67) and seven variables (including all the above factors except primary site) into the multivariate Cox regression model for CSS (AIC=755.65). **(Table 2)**

In the multivariate Cox analyses, with regard to OS, elder age (for every 1-year increase, Hazard Ratio (HR): 1.053, 95% Confidence Interval (95% CI): 1.040-1.066, *P*<0.001) and M1 classification (Referent: M0, HR: 6.218, 95% CI: 2.952-13.099, *P*<0.001) were associated with the highest mortality risk. On the other hand, with regard to CSS, elder age (for every 1-year increase, HR: 1.039, 95% CI: 1.023-1.055, *P*<0.001), high tumor grade (Referent: Low grade, HR: 12.484, 95% CI: 1.635-95.325, *P*=0.015) and M1 classification (Referent: M0, HR: 5.301, 95% CI: 2.287-12.288, *P*<0.001) were proved to be prognostic factors with the highest cancer-specific mortality risk. **(Table 2)**

**Figure 1:**
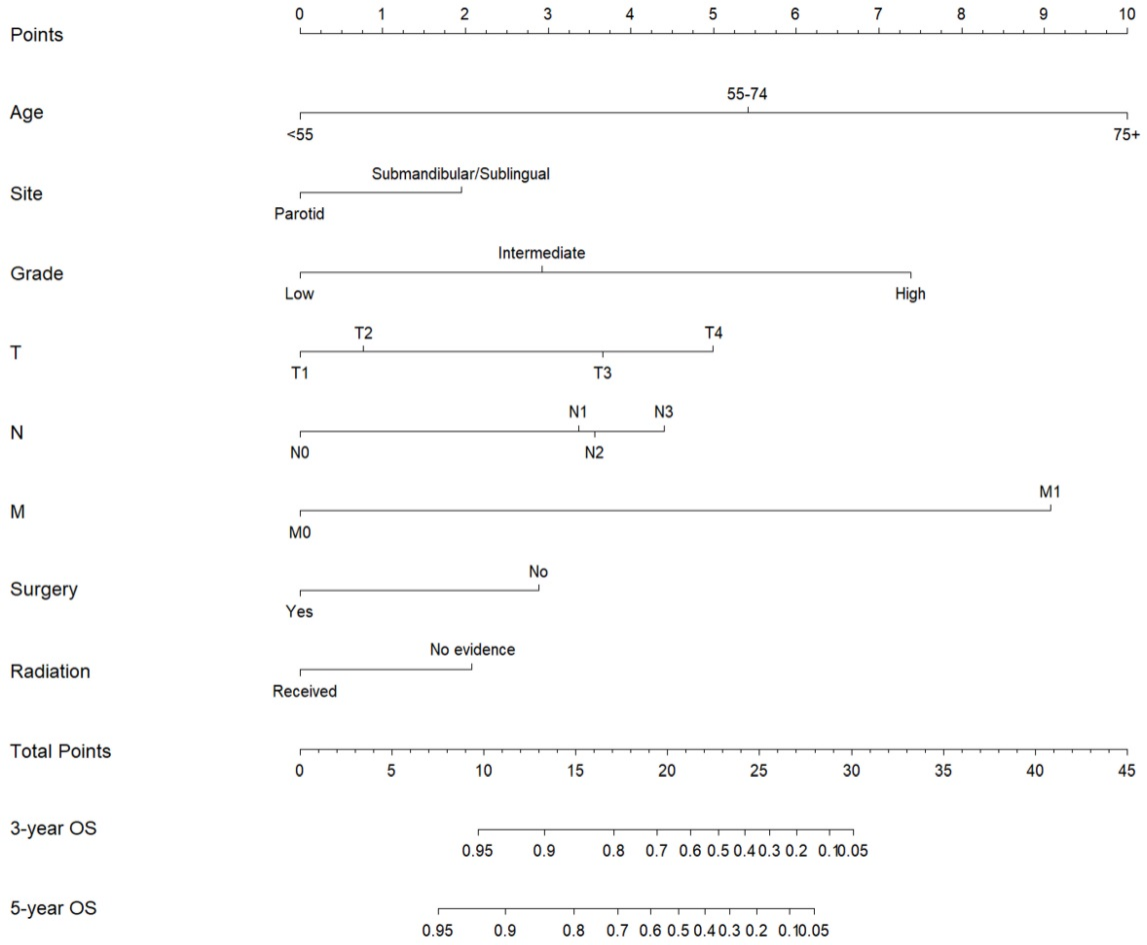
Nomogram estimating the 3- and 5-year overall survival of MaSG-MEC patients.

**Figure 2:**
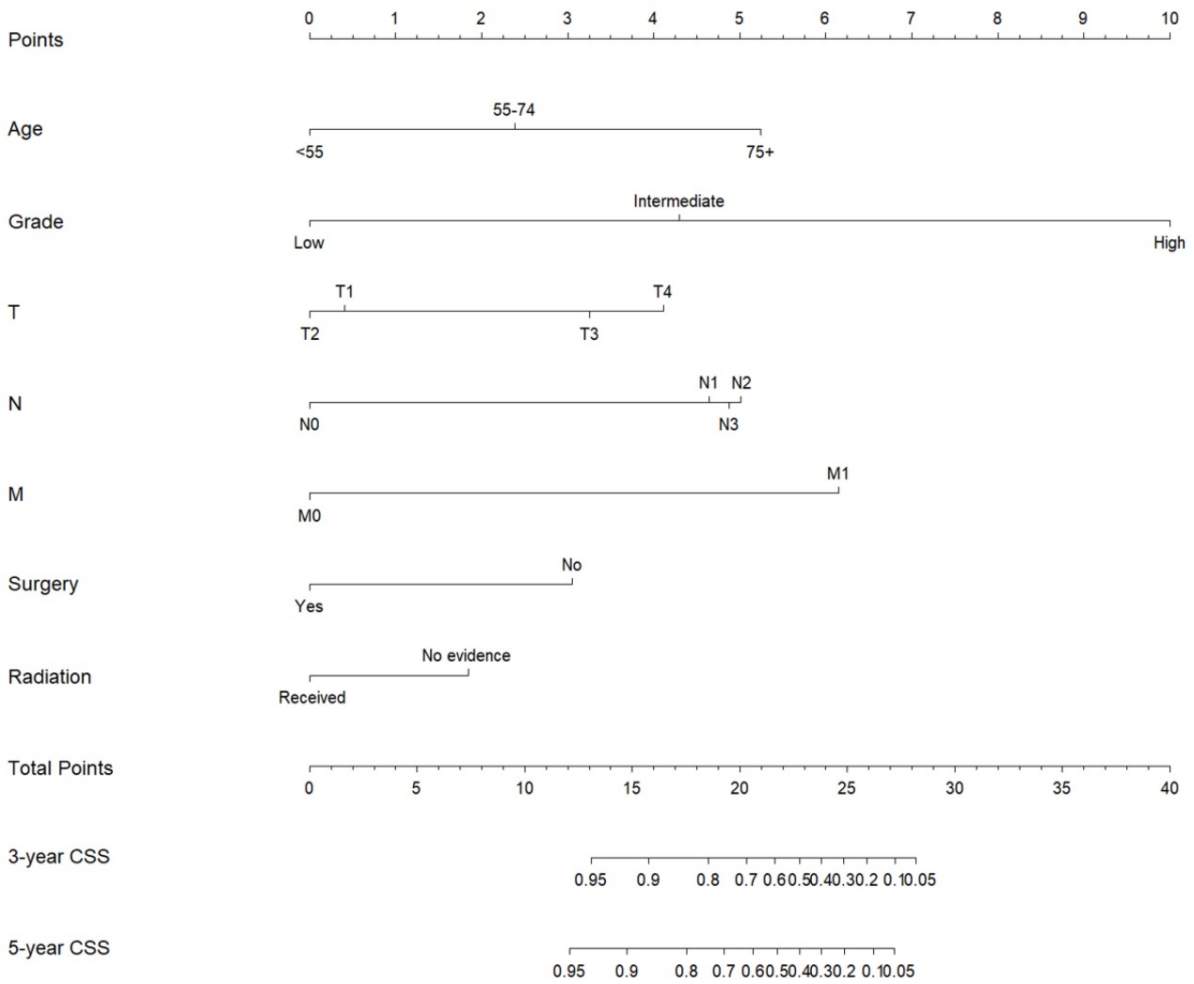
Nomogram estimating the 3- and 5-year cancer-specific survival of MaSG-MEC patients.

Subsequently, these clinicopathological variables were used to develop nomograms to predict the 3- and 5-year OS and CSS rates of MaSG-MEC patients **(Figure 1 and 2)**. Risk score of each predictor is summarized in **Table 3.**

It is comprehensible to use a nomogram and its corresponding risk score to estimate individualized survival. Take this case as an example, a 75-year-old male was diagnosed with high-grade left parotid mucoepidermoid carcinoma and underwent total parotidectomy. The final pathology report revealed a T2N3M0 tumor according to the 7th AJCC staging system, and the patient did not receive postoperative radiotherapy. To estimate his 3- and 5-year probabilities of OS and CSS, we should first look up to **Table 3** to obtain the risk scores of each predictor and add them up. He got 13.0 and 19.3 total points in the OS and CSS nomograms, accordingly. We then draw a vertical line from the Total Points scale to the 3- and 5-year OS or CSS scale in Figure 1 and obtained the corresponding survival rate. For this patient, his estimated 3- and 5- year OS rates were approximately 48% and 31%, respectively. In the same manner, the 3- and 5-year CSS rates were estimated as 59% and 49%, respectively.

### Performance of the nomograms

Our nomograms were internally and externally validated by the bootstrap method with 1000 resamples. In the internal validation *via* the training cohort, the C-indexes of the OS and CSS nomograms were 0.888 (95%CI 0.862-0.914) and 0.938 (95%CI 0.923-0.953), respectively, both of which were over 10% higher than the C-indexes of the 7th AJCC staging system [C-index: OS: 0.785 (95%CI 0.747-0.823), CSS: 0.821 (95%CI 0.788-0.854)]. Regarding the external validation *via* the validation cohort, the C-indexes of the nomograms [OS: 0.844 (95%CI 0.813-0.875), CSS: 0.882 (95%CI 0.854-0.910)] also showed superiority over the 7th AJCC staging system [OS: 0.743 (95%CI 0.699-0.786), CSS: 0.787 (95%CI 0.744-0.830)] and both the nomograms have improved the predictive accuracy by over 10%.

**Figure 3:**
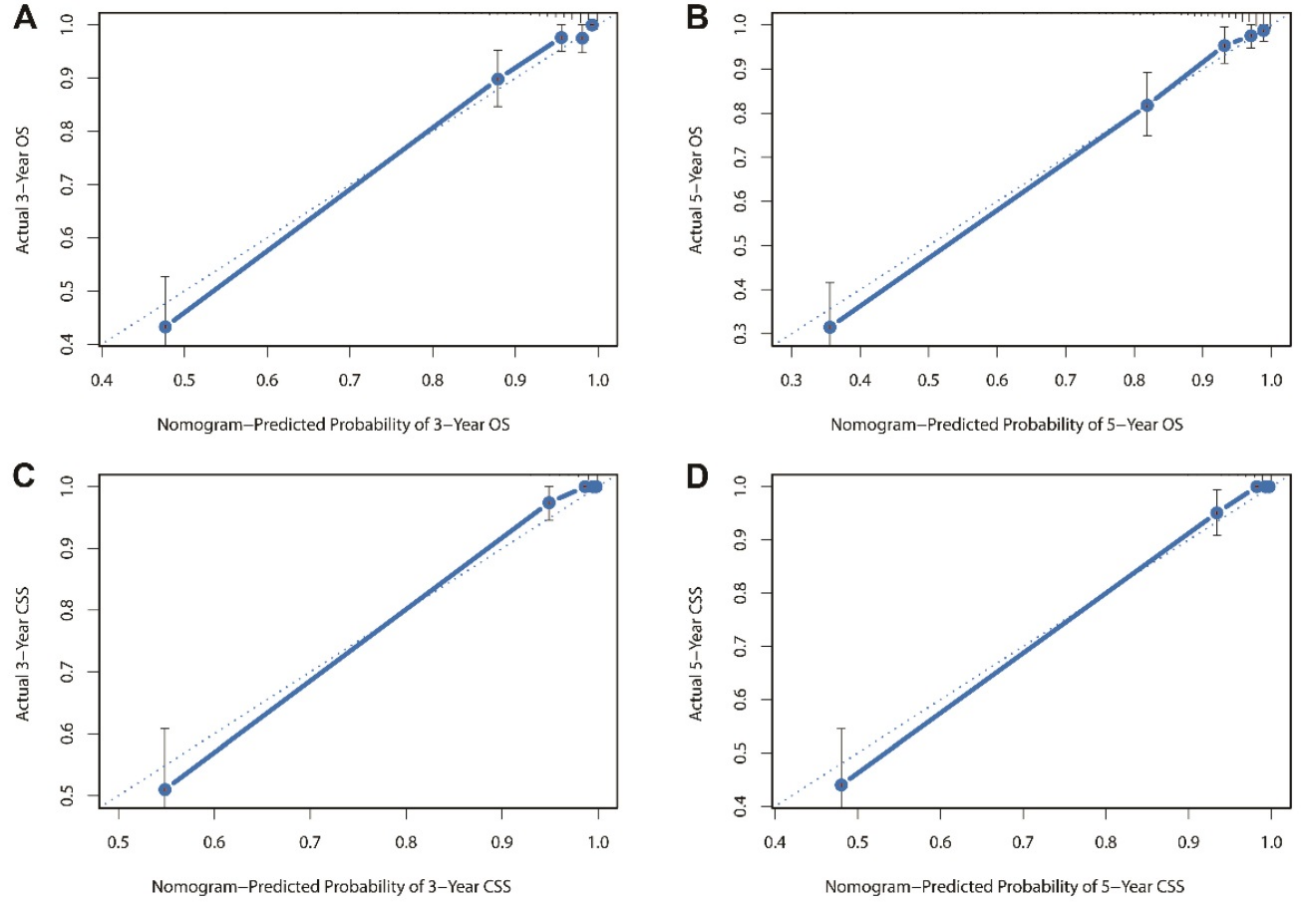
Internal calibration plots for (A) 3-year overall survival (B) 5-year overall survival (C) 3-year cancer-specific survival (D) 5-year cancer-specific survival.

**Figure 4:**
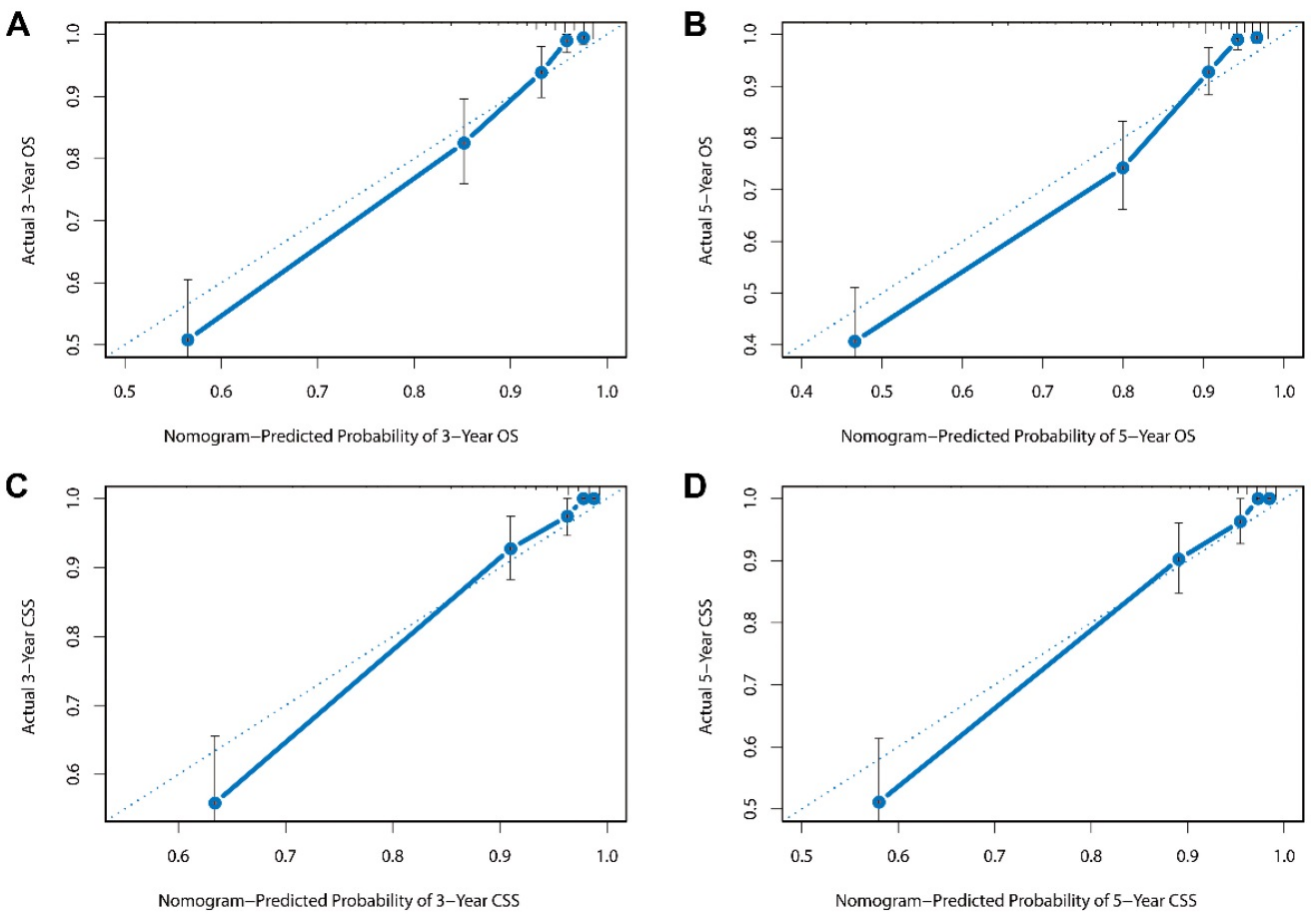
External calibration plots for (A) 3-year overall survival (B) 5-year overall survival (C) 3-year cancer-specific survival (D) 5-year cancer-specific survival.

Besides, the calibration plots in the internal and external validation are shown in **Figure 3** and **Figure 4**. In each calibration plot, the diagonal dashed line stands for perfect match between nomogram prediction (x-axis) and observed survival (y-axis). The training cohort was divided into 5 groups with equal sample size. The closer distances between the fit line and the diagonal line, the higher prediction accuracy the nomogram possessed. Consequently, both the internal and external calibration plots demonstrated excellent agreements between nomogram estimation and observed survival. **(Figure 3 and 4)**

## Discussion

Mucoepidermoid carcinoma is the most common type of malignancies originating from major salivary gland. Despite of its relatively low incidence, the clinical behavior of MEC differs tremendously, from slow growing indolent tumor with a generally good prognosis to aggressive tumor accompanied with distant metastasis and causing high mortality rate.^7^, ^17^, ^18^ Treatment strategies of MEC evolve as time goes by. In addition to radical resection of the primary tumor, patients with high risk factors are now generally recommended with postoperative radiation. Also, for those with locally advanced tumor or tumor accompanied with distant metastasis, systematic chemotherapy has also been included in the systematic treatment and novel tyrosine kinase inhibitors (TKI) is undergoing investigation. 19 It is undoubtedly of clinical significance to accurately predict the prognosis of patients with MaSG-MEC. However, the TNM system, which is widely used to estimate survival and making corresponding treatment decisions, can only reflect a few aspects of the tumor characteristics, not enough for the purpose of patient stratification and better choice of adjuvant therapy. Therefore, building a more effective predictive tool is rather necessary. Efforts have been made in seeking risk factors for MaSG-MEC over the past decades. Jegadeesh *et al.* reported that in parotid MEC, no adjuvant radiation and older age at diagnosis were associated with increased risk of local regional recurrence, underlining the essentiality of postoperative radiotherapy.^20^ Spellmen *et al.,* on the other hand, argued that in low-grade MEC, additional treatment had no impact on survival or recurrence.^17^ Inconsistencies like this arise widely among existent researches, challenging the management of MaSG-MEC.

Nomograms have been successfully constructed to predict the prognosis of many cancer types and are confirmed to be more accurate than the AJCC TNM staging system.^8^, ^15^, ^21^-^23^ Although Safina A et al. have successfully constructed a prognostic nomogram for MaSGC based on the data of 301 patients who underwent surgery at Memorial Sloan Kettering Cancer Centre (MSKCC),^24^ this study did not distinguish different histotypes in the nomograms, which somewhat reduced their practicability. Moreover, the single-centered research with a small sample size was not externally validated and its further application was likely to be limited. Similarly, Ju et al. also mixed different histotypes of MaSGC in their proposed nomograms without any distinction,^25^ which possibly led to considerable bias in the setting of a specific histology. To our knowledge, nomograms specifically focusing on the survival of MaSG-MEC have never been developed yet. Prospective approach towards this issue is rather consuming because of the rarity of MaSG-MEC. We therefore turned to retrospective data from the SEER database, and conduct our study using a large sample of MaSG-MEC patients.

In this study, we for the first time developed and validated prognostic nomograms for estimation of the 3- and 5-year overall and cancer-specific survival based on 1398 MaSG-MEC patients from the SEER program (699 in the training set and 699 in the validation set). Of note, using the training cohort, the C-indexes of the OS and CSS nomograms could achieve as high as 0.888 and 0.938, respectively, exceeding the TNM staging system by a large extent and thus indicating a higher reliability. More importantly, in the validation cohort, the excellent performance of the nomograms further verified the robustness in its capacity of prognostic prediction. The optimal agreements between the nomogram-based estimation and observed prognosis also ensured the repeatability.

Several clinicopathologic parameters have been identified in the present study to be independent prognostic factors for OS and CSS of MaSG-MEC, including age at diagnosis, primary site, tumor grade, primary site, T, N, M classification, surgery and radiotherapy. In the OS nomogram, age made the greatest contribution. In the CSS nomogram, on the other hand, tumor grade revealed a much stronger impact. High tumor grade was associated with a higher MEC-specific mortality risk than distant metastasis or locally advanced primary tumor. However, there is a lack of consensus regarding pathologic grading systems of MEC.^18^ In addition to the WHO system used in our study, the Armed Forces Institute of Pathology (AFIP) system,^26^ the modified Healy classification,^5^ and the Brandwein system^27^ are all more or less adopted in current clinical practice. Which is the best way to define tumor grade in MEC remains unsolved. More work is warranted to identify the optimal grading system to further increase the predictive efficacy of the nomograms. In both the OS and CSS nomograms, we also found that patients with T1 disease seemed to have a slightly higher risk score than those with T2 lesions, which is possibly due to the lack of records on some important clinicopathologic factors acting as confounders in the SEER database, such as vascular invasion, perineural invasion and surgical margins.

Using nomograms to estimate individual survival of MaSG-MEC patients has the following advantages. First, we are able to assess the proportion of 3- and 5-year overall survival and cancer-specific survival with a high accuracy. In clinical practice, it is feasible for us to formulate and adjust the follow-up strategy using the survival rate evaluated by our nomograms. For example, when facing a patient with a poor estimated prognosis, shorter follow-up intervals should be adopted in order to monitor the disease more effectively. Second, only eight commonly used variables are required to achieve a precise estimation with the help of the nomograms, making it easily accessible to all healthcare providers. Third, compared with the AJCC staging system, the better predictive performance of our nomograms may facilitate the choice of postoperative treatments, including radiotherapy, chemotherapy, and novel medication like tyrosine kinase inhibitors. Promising as these therapies seem to be, no solid improvement of related patient prognosis was observed to date. Our nomograms, as stated above, provide more accurate prognostic estimation of MaSG-MEC. Hence, we anticipate with precaution that they can serve as stratification criteria and consequently lead to a better understanding of the mechanism of the disease.

In our study, there are still some limitations inherent to the use of the SEER database. First, detailed information on chemotherapy and neck dissection is not available. To be more specific, after the year of 2003, SEER only collects the number of examined lymph nodes and the number of positive lymph nodes without any record of the surgical information (whether it is neck dissection, neck sampling or node biopsy). As a result, we are unable to incorporate chemotherapy and neck dissection into the nomograms. Second, important clinical manifestations such as surgical margins, perineural invasion, vascular invasion, and facial paralysis are not recorded in SEER, thus they are not included in our study. Third, status of comorbidities, recurrence and reoperation are not available in the SEER database as well. Fourth, there is some inevitable selection bias caused by its retrospective nature. For example, some patients were too sick or too frail to receive the treatment. Additionally, M1 disease and elder patients were less accessible to receive operation or radiotherapy. Limitation also comes with the methodology. Age was processed as a continuous variable. In this way, the impact of age would not be greatly underestimated by inadequacy of group stratification, nor would it be affected by the difference of cutoff values. However, it may cause slight overadjustment by extensive division of the age variable. This is an innate shortcoming of the methodology that cannot be completely eliminated.

In conclusion, based on the population-based SEER database, we have proposed and validated effective clinical nomograms to estimate overall survival and cancer-specific survival of patients with major salivary gland mucoepidermoid carcinoma. These nomograms might be useful to assist clinicians in predicting an individual's prognosis and planning treatment and follow-up schedules.

## Figures and Tables

**Table 1:** Patient demographics and baseline characteristics

Characteristics	Training cohort		Validation cohort		*P*
	n=699		n=699		
	No.	%	No.	%	
Age (median [mean±SD])	54 (52.4±19.4)		53 (52.5±19.5)		
Gender					0.109
Female	334	47.8	364	52.1	
Male	365	52.2	335	47.9	
Site					0.165
Parotid	622	89.0	605	86.6	
Submandibular/Sublingual	77	11.0	94	13.4	
Grade					0.253
Low	167	23.9	190	27.2	
Intermediate	341	48.8	313	44.8	
High	191	27.3	196	28.0	
T classification					0.994
T1	317	45.4	317	45.4	
T2	184	26.3	181	25.9	
T3	114	16.3	114	16.3	
T4	84	12.0	87	12.4	
N classification					0.457
N0	554	79.3	572	81.8	
N1	72	10.3	61	8.7	
N2	68	9.7	64	9.2	
N3	5	0.7	2	0.3	
M classification					0.293
M0	685	98.0	690	98.7	
M1	14	2.0	9	1.3	
Surgery					0.416
Yes	618	88.4	608	87.0	
No	81	11.6	91	13.0	
Radiotherapy					0.668
Received	336	48.1	328	46.9	
No evidence	363	51.9	371	53.1	

**Table 2:** Multivariate cox analyses of prognostic factors for overall and cancer-specific survival in the training cohort incorporating covariates identified by the smallest AIC value

Characteristics	Overall Survival		Cancer-specific Survival	
	HR (95%CI)	*P* value	HR (95%CI)	*P* value
Age (continuous variable)				
For every one year increase	1.053(1.040-1.066)	<0.001^***^	1.039(1.023-1.055)	<0.001^***^
Site				
Parotid	Reference		Not Included	
Submandibular/Sublingual	1.569(0.877-2.807)	0.129		
Grade				
Low	Reference		Reference	
Intermediate	1.765(0.776-4.016)	0.175	3.137(0.395-24.921)	0.280
High	3.687(1.602-8.483)	0.002^**^	12.484(1.635-95.325)	0.015^**^
T classification				
T1	Reference		Reference	
T2	0.880(0.498-1.554)	0.658	0.958(0.395-2.325)	0.924
T3	2.102(1.234-3.582)	0.006^**^	2.509(1.144-5.502)	0.022^*^
T4	2.607(1.581-4.298)	<0.001^***^	3.036(1.403-6.568)	0.005^**^
N classification				
N0	Reference		Reference	
N1	1.805(1.127-2.893)	0.005^**^	3.348(1.763-6.358)	<0.001^***^
N2	1.942(1.229-3.067)	0.003^**^	3.537(1.853-6.750)	<0.001^***^
N3	3.352(1.010-11.123)	0.048^*^	4.772(1.053-21.625)	0.043^*^
M classification				
M0	Reference		Reference	
M1	6.218(2.952-13.099)	<0.001^***^	5.301(2.287-12.288)	<0.001^***^
Surgery				
Yes	Reference		Reference	
No	1.664(0.996-2.781)	0.052	2.165(1.181-3.966)	0.012^*^
Radiotherapy				
Received	Reference		Reference	
No evidence	1.458(0.963-2.208)	0.074	1.603(0.886-2.899)	0.119

^*^ P <0.05; ^**^ P <0.01;^***^ P <0.001

**Table 3:** Predictive risk scores of each predictor in the nomograms

Characteristics	OS nomogram	CSS nomogram
Age (continuous variable)	Age (year)/10	Age (year)/10
For reference		
20	2	2
30	3	3
40	4	4
50	5	5
60	6	6
70	7	7
80	8	8
90	9	9
Site		
Parotid	0.0	Not Included
Submandibular/Sublingual	0.9	
Grade		
Low	0.0	0.0
Intermediate	1.1	3.0
High	2.5	6.6
T classification		
T1	0.3	1.0
T2	0.0	0.0
T3	1.7	2.5
T4	2.1	3.0
N classification		
N0	0.0	0.0
N1	1.1	3.1
N2	1.3	3.3
N3	2.3	4.0
M classification		
M0	0.0	0.0
M1	3.6	4.5
Surgery		
Yes	0.0	0.0
No	1.0	2.0
Radiotherapy		
Received	0.0	0.0
No evidence	0.7	1.2
